# Vagus Nerve Stimulation for Depression: A Systematic Review

**DOI:** 10.3389/fpsyg.2019.00064

**Published:** 2019-01-31

**Authors:** Hang Lv, Yan-hua Zhao, Jian-guo Chen, Dong-yan Wang, Hao Chen

**Affiliations:** ^1^College of Psychology, Nanjing University of Chinese Medicine, Nanjing, China; ^2^The First Medical College, Nanjing University of Chinese Medicine, Nanjing, China; ^3^Department of Medical Psychology, Nanjing Brian Hospital, Nanjing, China; ^4^The Second Medical College, Nanjing University of Chinese Medicine, Nanjing, China

**Keywords:** vagus nerve stimulation, depression, systematic review, evidence-based medicine, randomized controlled clinical trial

## Abstract

**Background:** Depression is a common mental disorder worldwide. Psychological treatments and antidepressant medication are the usual treatments for depression. However, a large proportion of patients with depression do not respond to the treatments. In 2005, Vagus nerve stimulation was approved for the adjunctive long-term treatment of chronic or recurrent depression in adult patients experiencing a major depressive episode who had failed to respond to four or more adequate antidepressant treatments. However, the efficacy of VNS for treating depression remains unclear. Accordingly, we performed a systematic review to evaluate the efficacy and safety of VNS.

**Methods:** We conducted a systematic review in accordance with the Cochrane Handbook for Systematic Reviews of Interventions. Systematic search was performed in the database of Pubmed, Embase, CENTRAL, and Web of science for identifying the suitable trials. Suicidal rate was considered as the primary outcome in this review.

**Result:** Only two randomized sham controlled add-on studies including 255 cases (134 with VNS treatment and 121 control cases) were included in this review. None of the studies reported suicidal rate. We performed a qualitative analysis and it is suggested that there was no significant statistic difference between VNS and sham VNS on the score of 24-item Hamilton Rating Scale for Depression (HAMD_24_) (MD: −2.40, 95% CI: −7.90 to 3.10). Similar findings were also reported on improvement percentage of HAMD_24_ (MD: 1.00, 95%CI: −6.06 to 8.06), Montgomery-Asberg Depression Rating Scale (MADRS) (MD: 4.70, 95%CI: −2.98 to 12.38) and 30 item Inventory of Depressive Symptomalogy-Self-Report (IDS-SR_30_) (MD: 4.9, 95%CI: −1.89 to 11.69). However, a marginal difference of Beck Depression Inventory self-rating score was detected between the real and sham treatment (MD: 7.80, 95% CI: 0.34 to 15.26). Aminor effect of IDS-SR_30_was also found in real VNS group (RR: 2.33, 95% CI: 1.07 to 5.10).

**Conclusion:** The efficacy and safety of VNS for depression is still unclear. Further randomized controlled trials are needed to confirm the efficacy and safety of VNS.

## Introduction

Depression is a common mental disorder worldwide, with more than 300 million people affected. At its worst, depression could lead to suicide. Close to 800 000 people die due to suicide every year (WHO, 2018). Psychological treatments and antidepressant medication are the usual treatment for depression. However, 20 to 40% of patients with major depressive disorder do not show substantial clinical improvement on their first treatment with antidepressant medication (Fava and Davidson, 1996; Sackeim, 2001; Rush et al., 2006). Moreover, medications, including antidepressants, are often associated with significant side effects, for example, metabolic abnormalities and sexual dysfunction (Kupfer et al., 2012).

In 1997, vagus nerve stimulation (VNS) therapy which comprises an implanted electrical pulse generator to stimulate the vagus nerve was approved by the United States Food and Drug Administration (FDA) as an adjunctive therapy for reducing the frequency of seizures in adults and adolescents who were refractory to antiepileptic medications (Schachter, 2002). In 2005, it was further approved as the adjunctive long-term treatment for patients with chronic or recurrent depression who experienced a major depressive episode and failed to respond to four or more adequate antidepressant treatments (Helmers et al., 2012; Berry et al., 2013). As the best way for providing evidence for clinical practice, several systematic reviews have been performed for evaluating the exact efficacy and safety of VNS for depression (Daban et al., 2008; Martin and Martín-Sánchez, 2012; Berry et al., 2013; McGirr and Berlim, 2018). However, the findings of these reviews were inconclusive and did not update in time. Thus, it is the right time to identify the latter primary trials to confirm the efficacy of VNS in treating depression.

Systematic review is a best approach of providing evidence for clinical practice. Although VNS was approved as the adjunctive long-term treatment for chronic or recurrent depression by FDA (Helmers et al., 2012; Berry et al., 2013), there is still no sufficient evidence to confirm the efficacy of this treatment To supply the evidence of VNS in treating depression, we performed a systematic review to ascertain the technique's efficacy and safety on the basis of available the evidence.

## Methods

### Systematic Review Details

The systematic review was performed in accordance with the Cochrane Handbook for Systematic Reviews of Interventions (Higgins, 2011).

### Study Design

We enrolled randomized controlled trials (RCTs) that were published in formal English journals.

### Participants, Interventions, Comparators

Patients diagnosed as primary diagnosis of major depressive disorder or bipolar I or II disorder were involved in this review. Interventions should be Vagus nerve stimulation (VNS) or VNS combined with treatment as usual. Comparators should be usual treatment as or sham VNS.

### Search Strategy

JGC and DYW systematically searched the PUBMED, EMBASE, Cochrane Library, Web of science, Chinese National Knowledge Infrastructure (CNKI) and Wan-fang databases from inception to 6th Sep, 2018 with MeSH terms and key words without language restrictions. The search terms were (“depressive disorder” OR depression OR melancholia OR dysthymic OR bipolar) AND (vagus OR vagus nerve stimulation) AND (randomized controlled trial OR controlled clinical trial OR randomized OR clinical trials). We also checked the reference lists of relevant reviews and included trials to identify further studies that meet the inclusion criteria for this systematic review.

### Data Sources, Study Sections, and Data Extraction

Two reviewers (JGC and DYW) screened all the literature and extracted data independently using a standardized form. This was pre-designed for collecting information on trial characteristics such as first author, language, number of patients, mean age of the patients, diagnostic criteria, grades of hypertension, acupuncture treatment, control types, sessions of treatment, treatment course and outcome measures. Disagreements were resolved in consultation with the third reviewer (HL).

## Outcomes

### Primary Outcomes

Suicide is the most serious consequence of depression. Therefore, suicide rate was considered as the primary outcome in this review.

### Secondary Outcomes

Twenty-four item Hamilton Rating Scale for Depression (HAMD_24_), Beck Depression Inventory self-rating score (BID), Montgomery-Asberg Depression Rating Scale (MADRS), Clinical Global Improvement ratings (CGI-I), 30 item Inventory of Depressive Symptomalogy-Self-Report (IDS-SR_30_), response rate based on different scale score and adverse effect were listed as the secondary outcomes.

### Risk of Bias Assessment

Two reviewers (YHZ and HC) assessed the risk of bias using the Cochrane Collaboration's tool for assessing risk of bias (Zeng et al., 2015). Each trial was scored as high, low or unclear risk for the following 7 domains: (WHO, 2018) random sequence generation (selection bias); (Fava and Davidson, 1996) allocation concealment (selection bias); (Rush et al., 2006) blinding of participants and personnel (performance bias); (Sackeim, 2001) blinding of outcome assessment (detection bias); (Kupfer et al., 2012) incomplete outcome data (attrition bias); (Schachter, 2002) selective reporting (reporting bias); (Berry et al., 2013) other bias. Disagreements were resolved in consultation with the third reviewer (HL).

### Data Analysis

Continuous data (such as score assessments) was presented as mean differences (MDs) with 95% confidence interval (CI), whereas, dichotomous data (such as response rate) was presented as relative risk (RR) with 95% CI. Statistical heterogeneity across trials was assessed by the Cochran *Q*-test (*P* < 0.1 for statistical significance) and quantified by the *I*^2^ statistic. According to the Cochrane Handbook for Systematic Reviews of Interventions (Version 5.10), *I*^2^ > 50% was defined as significant heterogeneity. Heterogeneous data was pooled using the random-effects model. Publication bias was evaluated by visually inspecting a funnel plot if more than 9 studies was included. Meta-analysis was performed in the case of more than three homogeneity studies by RevMan 5.3 software.

## Result

### Study Selection

**Figure 1:**
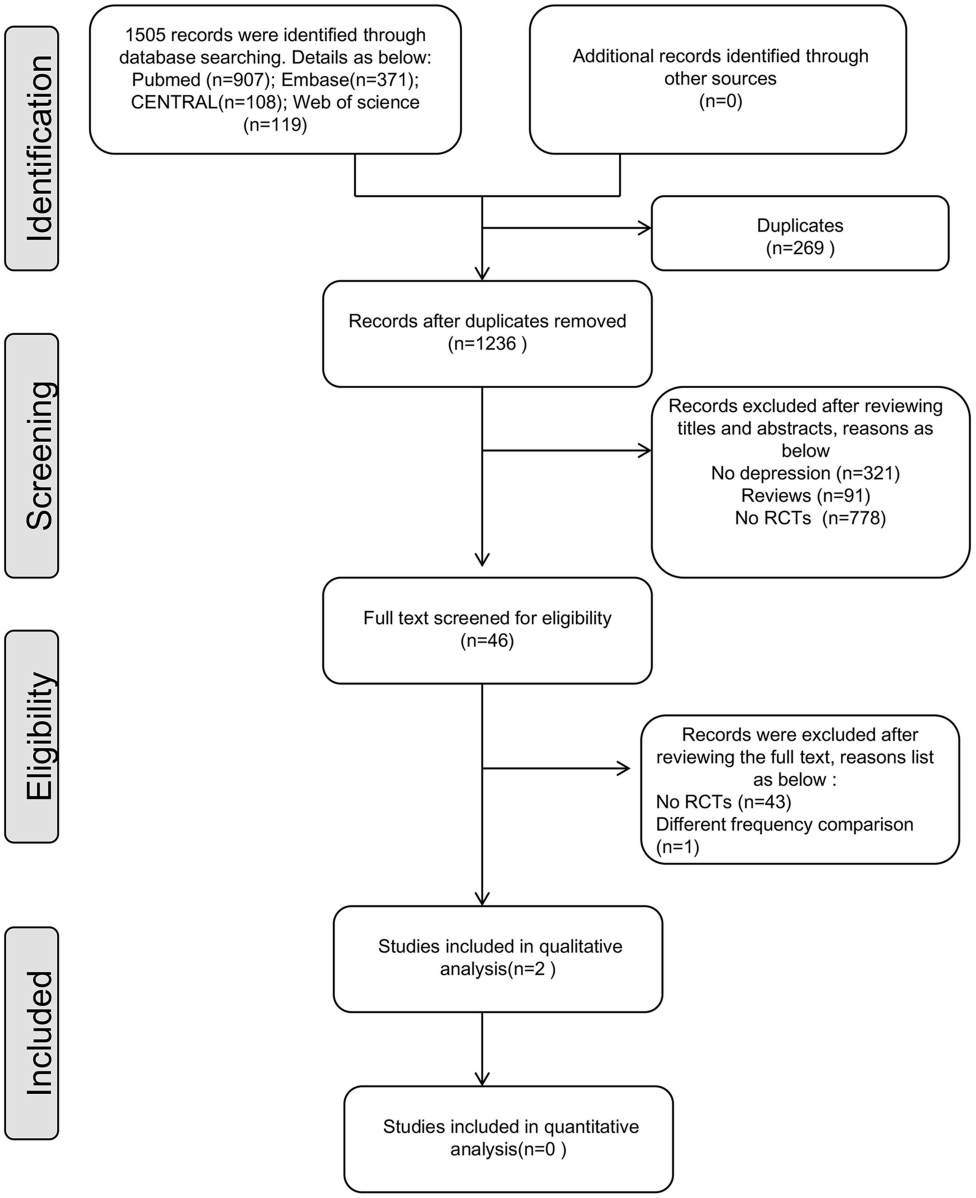
Flow chart of randomized controlled trial selection (based on PRISMA).

Figure 1 shows the flow chart of the study selection process based on PRISMA guidelines (Figure 1). One thousand five hundred and five records were identified during the initial search. After removing the duplicate records and screening the full text, 2 trials were included in this review (Rush et al., 2008; Hein et al., 2013).

### Characteristics of the Included Studies

These two trials were published in 2005 and 2013, respectively, both of which were randomized sham controlled add-on studies with two arms. Two hundred and fifty-five patients (134 in VNS group and 121 in control group) were included in the final analysis. One trial used a non-invasive method of stimulating the vagus nerve on the outer canal of the ear (Hein et al., 2013), while the other one used the implantation device (Rush et al., 2008).

The suicidal rate was not evaluated in these two trials. HRSD_24_ was recoded in both two trials. It was presented as the sore in one trial. In the other trial, HRSD_24_ response rate was presented. which was defined as ≥50%reductionafter 10 weeks treatment at the baseline (Rush et al., 2008).

Beck Depression Inventory self-rating score was considered as a secondary outcome in Hein's study (Hein et al., 2013), while Rush et al. took MADRS, CGI-I, and IDS-SR_30_ as the secondary outcomes (Rush et al., 2008). Adverse effects were recorded in both two trials. More detail information was presented in Table 1.

**Table 1:** Characteristics of the included studies.

**ID**	**Participants**	**Intervention**		**No. of patients for evaluation**			**Course**	**Outcome**
		**Treatment**	**Control**	**Treatment**	**Control**	**Total**		
Rush J 2005 (Rush et al., 2008)	Major depressive disorder or bipolar I or II disorder.	Invasive VNS + TAU	Sham VNS + TAU	112	110	222	10 Weeks	1,2,3,4,5,6,7
Hein E 2013 (Hein et al., 2013)	Major depressive episode.	None-invasive VNS + TAU	Sham VNS + TAU	22	11	33	2 Weeks	8,9

*VNS, vagus nerve stimulation; TAU, treatment as usual; Outcomes, 1 = HRSD_24_ Response Rate; 2 = MADRS Response Rate; 3 = CGI-I Response Rate; 4 = IDS-SR_30_ Response Rate, 5 = Percentage of HRSD_24_ Improvement from Baseline; 6 = Percentage of MADRS Improvement from Baseline; 7 = Percentage of IDS-SR_30_ Improvement from Baseline; 8 = HRSD_24_ score change; 9 = Beck Depression Inventory self-rating score change; HAMD_24_, 24-item Hamilton Rating Scale for Depression, MADRS, Montgomery-Asberg Depression Rating Scale, CGI-I, Clinical Global Improvement ratings, IDS-SR_30_, 30 item Inventory of Depressive Symptomalogy-Self-Report*.

### Risk of Bias of the Included Studies

**Figure 2:**
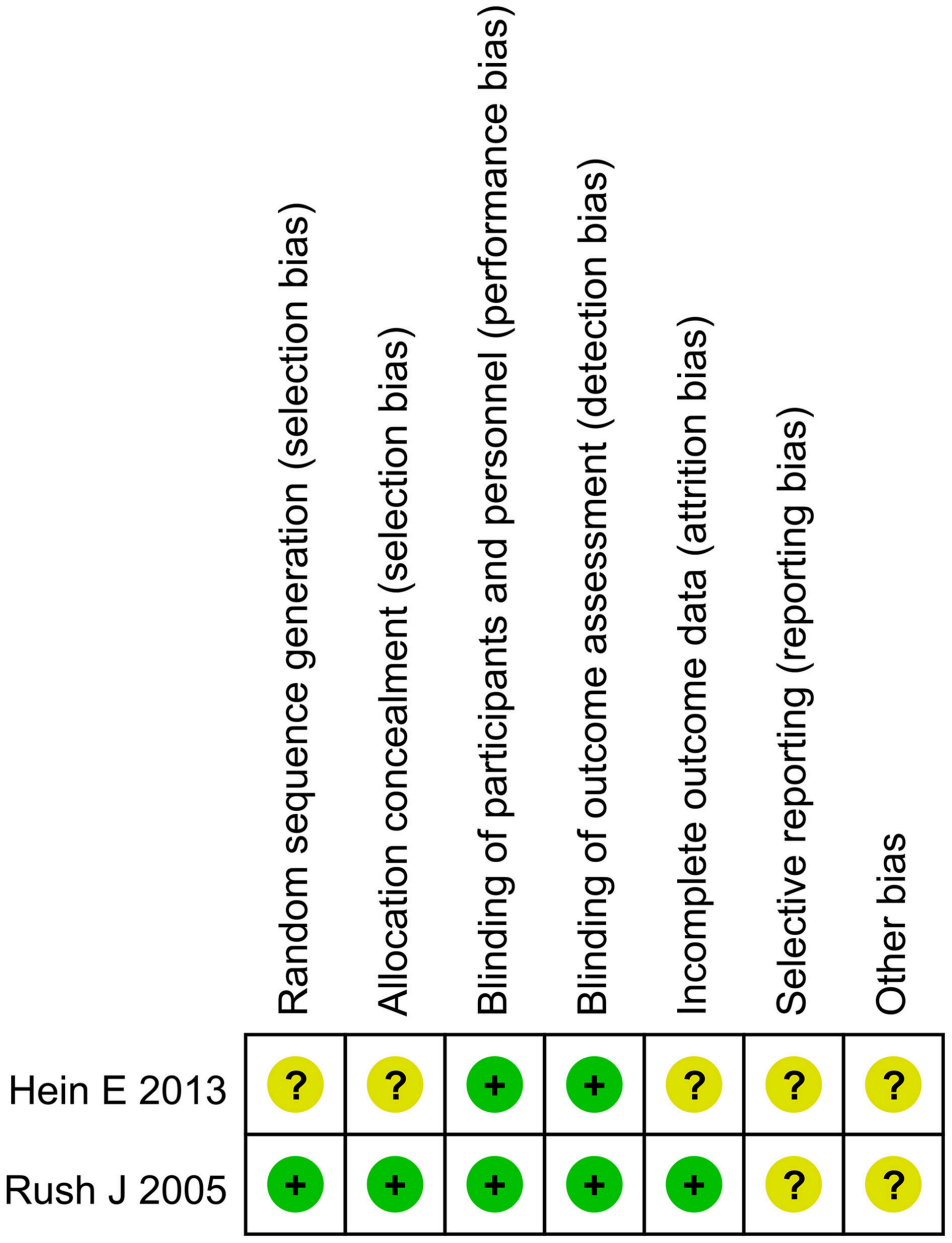
Risk of bias graph of the included trials.

As shown in Figure 2, the two trials were all double blind design. Rush et al. described the random procedure in detail and took Last Observation Carried Forward (LOCF) for the final statistical analysis (Rush et al., 2008), while Hein et al. did not (Hein et al., 2013). Selective reporting was unclear in both two studies for all the included studies as we have no access to the study protocol.

### Qualitative Findings

We did not pool the results of the two trials because of the potential heterogeneity between the two studies.

### Score of the Scale Assessment

**Figure 3:**
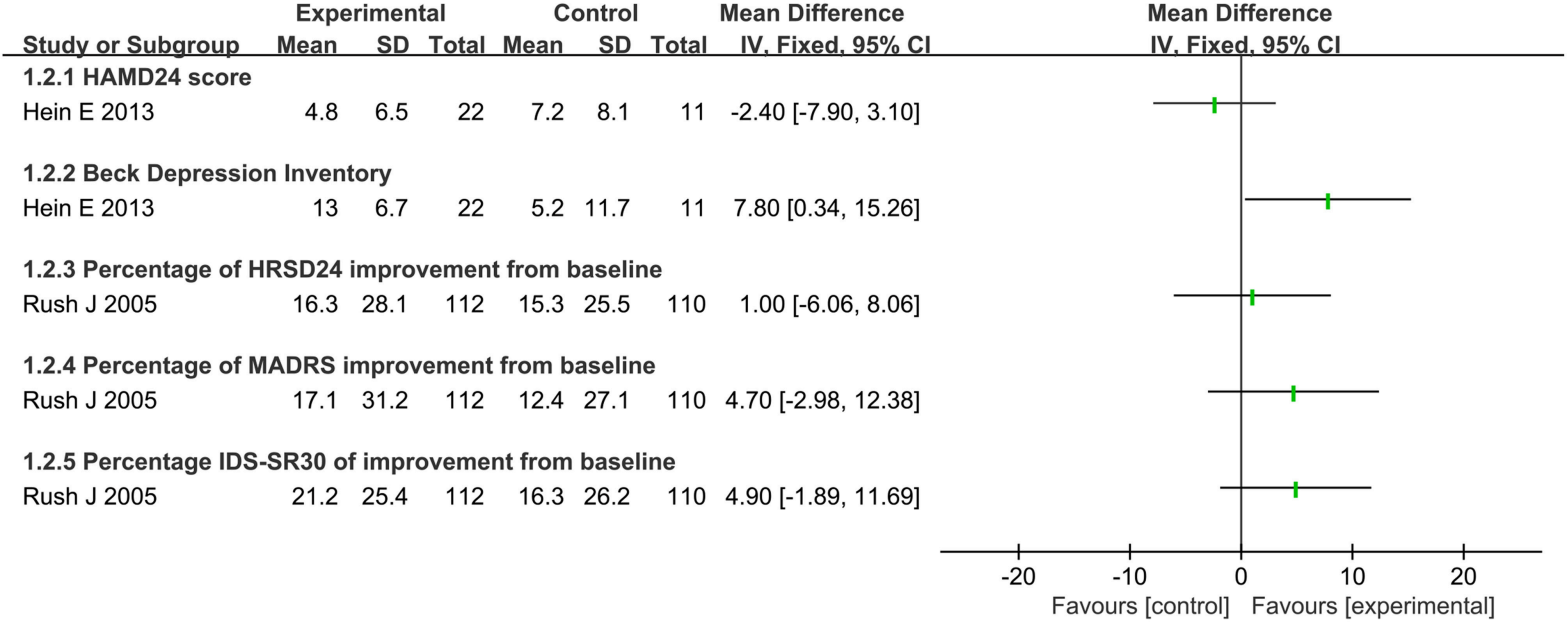
Summary of findings on score of the scale assessment.

**Figure 4:**
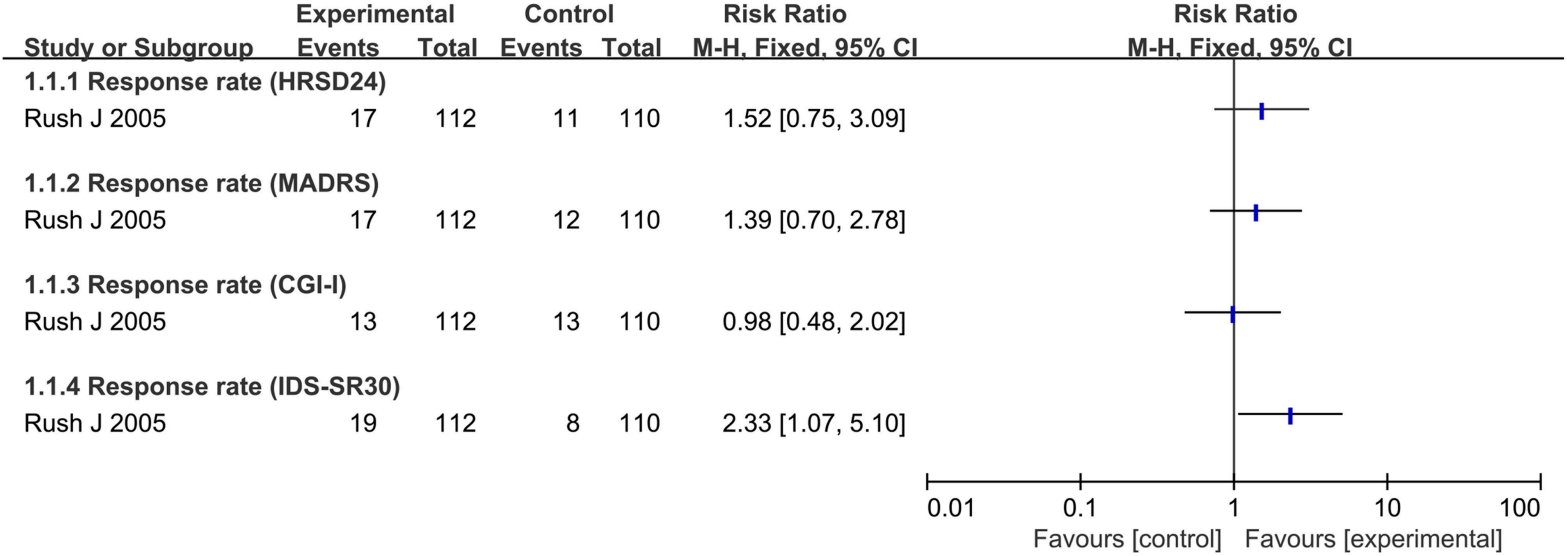
Summary of findings on response rate.

There was no significant difference between VNS and sham VNS on the score change of HAMD_24_(MD: −2.40, 95% CI: −7.90 to 3.10) (Figures 3, 4). Similar findings were also reported on improvement percentage of HAMD_24_ (MD:1.00, 95%CI: −6.06 to 8.06), MADRS(MD: 4.70, 95%CI: −2.98 to 12.38) and IDS–SR_30_(MD: 4.9, 95%CI: −1.89 to 11.69). Significant difference of Beck Depression Inventory self-rating score was detected between the real and sham treatment (MD: 7.80, 95%CI: 0.34 to 15.26).

### Response Rate

Only one trial focused on the response rate based on different scale score (Rush et al., 2008). Most of the response rate showed no significant difference between real VNS and sham treatment except for IDS-SR_30_, which showed a better effect in real VNS group (RR: 2.33, 95% CI: 1.07 to 5.10)_._

### Safety Evaluation

Hein et al. reported that no adverse side effects were observed during the whole course of treatment and after the trial (Hein et al., 2013). Rush et al. reported that voice alteration was the most common adverse effect, which was occurred in 16 cases treated with VNS and 14 cased with sham treatment (Rush et al., 2008).

### Publication Bias

We did not perform publication bias evaluation as only two trials were included in this review. However, we inferred that publication bias would be exist as the small sample size and the unstable findings of the included studies.

## Discussion

### Summary Findings of the Review

Depression is a common mental disorder worldwide, which could lead to suicide (WHO, 2018). Thus, suicide rate may be the most important final endpoint for the effect evaluation of VNS in treating depression. Based on this clinical setting, suicidal rate was considered as the primary outcome. Unfortunately, only two RCTs were identified and suicide rate was not reported in both trials. We had to focus on the secondary outcomes such as HAMD24 sore, improvement percentage of HAMD_24_, MADRS, IDS–SR_30_ and response rate.

According to the findings, there may be no significant difference of main outcomes of depression between VNS and sham treatment. As lack of the primary outcome and the small sample size of the included studies, this review did not yield a stable and definitive evidence of efficacy of VNS in treating depression.

To identify the exact efficacy of VNS for depression, several systematic reviews (Daban et al., 2008; Martin and Martín-Sánchez, 2012; Berry et al., 2013; McGirr and Berlim, 2018) have been published on the same topic and one of them is an overview of review (McGirr and Berlim, 2018). Compared with the current review, these reviews only considered the Scale scores as the primary outcomes. In addition, some of the reviews involved RCTs and Non-RCTs which may lead to methodological heterogeneity in the analysis. Furthermore, most of the reviews were outdated and should be update (Daban et al., 2008; Martin and Martín-Sánchez, 2012; Berry et al., 2013). All of these may downgrade the quality and applicability of the reviews (Table 2).

**Table 2:** Comparisons between the published reviews and the current one.

**References**	**Clinical settings**	**No. of trials**	**Search date**	**Outcomes**	**Findings**
Berry et al., 2013	VNS for treatment-resistant	A patient-level meta-analysis	Not applicable	MADRS, CGI-I	VNS + TAU has greater response and remission rates that are more likely to persist than TAU.
Daban et al., 2008	VNS for treatment-resistant depression	18	Sep. 2007	HDRS, IDS-SR, MADRAS, Responder rate, remitted rate, CGI-I, CGI-SI, GAF, SF-36	VNS may be an new approach for TRD, further clinical trials are needed to confirm its efficacy in major depression.
Martin and Martín-Sánchez, 2012	VNS for depression	14	Dec. 2010	Level of depression, percentage of responders	Insufficient data are available to describe VNS as effective in the treatment of depression.
McGirr and Berlim, 2018	Thearpeutic neuromodulation for major depression	An overview of review	Nov. 2017	MADRS, CGI-I	Robustness evidence associated with VNS for major depression is inferior to that associated with non-invasive neuromodulation approaches.
The present one	VNS for depression	10	Sep. 2018	HAMD_24_, MADRS, IDS-SR_30_, Beck Depression Inventory self-rating score	The efficiency and safety of VNS for depression is still unclear, there is no sufficient evidence for VNS in treating depression

*VNS, Vagus nerve stimulation; MADRS, Montgomery-Asberg Depression Rating Scale; CGI-I, Clinical Global Impressions scale's Improvement subscale; HDRS/HAMD_24_, Hamilton Depression Rating Scale; IDS-SR, Inventory of Depressive Symptoms; CGI-SI, Clinical Global Impressions Severity Index; GAF, Global Assessment of Function; SF-36, The Short Form (36) Health Survey*.

### Limitations of the Study

Lack of sufficient primary trials and long-term follow-up data may narrow our findings and indicate high variability.

### Implications for Clinicians

Although VNS is a novel approach for treating depression and has been approved by FDA, it still lacks sufficient evidence to confirm its efficacy and safety. Moreover, the potential mechanism and the cost-effectiveness is still unknown. Clinicians should take VNS as the treatment for depression according to the clinical settings.

### Future Perspectives

VNS seems to be a new approach for depression treatment. However, its efficacy and safety needs to be further investigated. Suicidal rate is necessary to be evaluated in RCTs.

## Conclusion

The efficacy and safety of VNS for depression treatment is still unclear.

## Author Contributions

HL and HC conceived the study. JC and DW performed the literature search and extracted the data. YZ and HC assessed risk of bias. HC performed statistical analysis. HL and YZ drafted the manuscript. All the authors critically revised the manuscript. HL had full access to all of the data in the study, and took responsibility for the integrity of the data and the accuracy of the data analysis.

### Conflict of Interest Statement

The authors declare that the research was conducted in the absence of any commercial or financial relationships that could be construed as a potential conflict of interest.
